# The Dual Nature of Sinoatrial Node Remodelling in Athletes: A Systematic Review of Electrophysiological Adaptations and the Pathological Tipping Point

**DOI:** 10.3390/ijms262412052

**Published:** 2025-12-15

**Authors:** Liang Yue, Jiaying Li, Hui Wang, Shuang Li, Henggui Zhang

**Affiliations:** 1 Key Laboratory of Medical Electrophysiology, Ministry of Education and Medical Electrophysiological Key Laboratory of Sichuan Province (Collaborative Innovation Center for Prevention of Cardiovascular Diseases), Institute of Cardiovascular Research, Southwest Medical University, Luzhou 646000, China; y18634453772@163.com (L.Y.); 18980682349@163.com (J.L.); wh526h@163.com (H.W.); shuanglee111u@163.com (S.L.); 2 Biological Physics Group, Department of Physics and Astronomy, The University of Manchester, Manchester M13 9PL, UK; 3 Beijing Academy of Artificial Intelligence, Beijing 100084, China

**Keywords:** exercise training, cardiac remodelling, sinoatrial node, SAN dysfunction, exercise training, ion channels, autonomic nerves

## Abstract

The “athlete’s heart” phenotype, featuring resting bradycardia, has traditionally been viewed as a benign adaptation. However, emerging evidence associates prolonged, high-intensity endurance training with an increased risk of clinical sinoatrial node dysfunction. This systematic review synthesizes evidence on exercise-induced intrinsic Sinoatrial Node (SAN) electrophysiological remodelling and evaluates its dual nature along the adaptation–pathology continuum. Following PRISMA guidelines, a systematic search of PubMed, Web of Science, and Google Scholar (2000–2025) identified 17 eligible studies. Analysis revealed that in humans, rodents, and rabbits, exercise induces intrinsic SAN electrophysiological remodelling—a “membrane clock” reset characterized by coordinated downregulation of pacemaker currents, notably Hyperpolarization-activated cyclic nucleotide-gated cation channel (I_f_), via the Nkx2.5-miR-423-5p transcription factor pathway. Evidence for “calcium clock” involvement remains inconsistent. In contrast, large animal models (e.g., dogs, horses) show only parasympathetic-mediated bradycardia without intrinsic remodelling. Training loads may induce structural changes (e.g., fibrosis), providing an anatomical substrate for pathology. Moderating factors such as training type and ageing contribute to a phenotype of “acquired SAN reserve reduction. Exercise-induced intrinsic SAN remodelling is a physiological adaptation mechanism that, under certain conditions, can cross a threshold to become a pathological cause of clinical dysfunction. Recognizing this continuum is essential for risk stratification and future therapeutic innovation.

## 1. Introduction

Exercise training induces a spectrum of cardiac structural and functional adaptations, collectively termed the “athlete’s heart” [1,2]. A classic electrocardiographic finding in athletes is resting bradycardia, traditionally viewed as a benign marker of heightened vagal tone and superior cardiac efficiency [3,4]. However, emerging clinical and experimental evidence indicates that exercise training may be associated with an elevated risk of clinically significant sinoatrial node (SAN) dysfunction, manifesting as profound symptomatic bradycardia, sinus pauses, and even the necessity for permanent pacemaker implantation [5,6,7].

Although athletic bradycardia was once primarily attributed to shifts in autonomic nervous system regulation, research over the past two decades, however, has firmly established that exercise-induced SAN itself undergoes significant intrinsic electrophysiological and structural remodelling. This remodelling involves the key cellular mechanisms governing spontaneous diastolic depolarization—the “membrane clock” and “calcium clock.” Specifically, the adaptations occur in the hyperpolarization-activated cyclic nucleotide-gated (HCN) channel current (I_f_), the L-type calcium current (I_Ca-L_), and the function of ryanodine receptors (RyR2) in sarcoplasmic reticulum calcium cycling [8,9,10]. While these molecular adaptations are increasingly well-characterized, a critical and unresolved question persists: to what extent does this intrinsic remodelling represent a purely physiological adaptation, and under what conditions might it cross a threshold to become a substrate for pathology?

Current evidence remains fragmented across disparate animal models and limited human studies, lacking a cohesive mechanistic framework. The “tipping point” from adaptation to pathology is poorly defined, and the influence of potential moderators such as species, training modality, and age on the remodelling phenotype remains inadequately explored. This systematic review therefore aims to: (1) synthesize the relative contributions of autonomic regulation and intrinsic SAN remodelling to exercise-induced bradycardia; (2) systematically review on the effects of exercise training on the intrinsic electrophysiological properties of the SAN, focusing on the “membrane clock” and “calcium clock” mechanisms; and (3) critically examine the dual nature of this electrophysiological remodelling—its physiological boundaries and its potential progression to pathological states, thereby providing a novel perspective on the etiology of impaired SAN function in athletes.

## 2. Evidence Synthesis

This systematic review synthesizes evidence on the risk factors that modulate the transition from adaptive to pathological states, characterizing exercise-induced SAN remodelling as a complex process spanning intrinsic electrophysiological and structural alterations. However, the field is complicated by significant interspecies differences and a poorly defined boundary between adaptive and pathological remodelling. Key findings are detailed in Table 1.

## 3. Divergent Mechanisms of Bradycardia

There is currently no consensus regarding the fundamental mechanisms underlying sinus bradycardia in athletes. Ambulatory monitoring data corroborate the presence of a non-autonomic mechanism for heart rate reduction [23]. The dissociation between profound bradycardia and unaltered heart rate variability (HRV) in elite athletes further indicates an intrinsic downregulation of the SAN pacemaker mechanism [17]. This challenges the prevailing explanation that attributes increased HRV in athletes solely to adaptive neurocardiac regulation [24]. Instead, robust biophysical evidence identifies the basal heart rate as the primary determinant of HRV [25,26]. Consequently, the observed increase in HRV in athletes is likely a consequence of intrinsic bradycardia rather than an independent index of elevated vagal tone [25]. Direct evidence for intrinsic SAN remodelling in both humans and animal models (such as rabbits and rodents) includes a persistently lower intrinsic heart rate and a prolonged sinus node recovery time, as observed in isolated tissue preparations and in vivo studies following complete autonomic blockade [3,10,16,21,22]. In contrast, studies in mice, horses and dogs attribute bradycardia solely to enhanced parasympathetic tone, revealing no intrinsic SAN changes [4,14,20]. These fundamental disparities highlighted by cross-species comparisons underscore the necessity for caution when extrapolating findings from animal models to humans.

## 4. Electrophysiological Mechanisms of Exercise-Induced Sinus Node Remodelling

### 4.1. Membrane Clock Remodelling: Coordinated Downregulation of Pacemaker Currents

The most consistent ionic correlate of exercise-induced bradycardia in both humans and rodents is downregulation of the Hyperpolarization-activated Cyclic nucleotide-gated channel 4 (HCN_4_) channel and its corresponding I_f_ current. Patch-clamp analysis documents that training reduces I_f_ current density by 40–47% (measured at –105 mV) within the pacemaking potential range [8,9]. This finding is functionally corroborated in human athletes, as evidenced by their blunted heart rate response to the I_f_-specific blocker ivabradine, alongside more pronounced intrinsic bradycardia under complete autonomic blockade [10]. Notably, this form of HCN_4_ downregulation is reversible following exercise cessation [8].

Mechanistically, this process is governed by a precise post-transcriptional pathway. The transcription factor Nkx2.5 drives the expression of microRNA-423-5p, which subsequently binds to the 3′-UTR of HCN_4_ mRNA, inhibiting its translation. Critically, administration of a miR-423-5p antagonist in vivo reverses HCN_4_ downregulation, restores I_f_ current density, and normalizes heart rate [10]. It is important to note, however, that this pattern of HCN_4_ downregulation is not universal across all species. Studies in canine models have reported an increase in HCN_4_ protein levels following training, highlighting a fundamental interspecies discrepancy in the adaptive response of the pacemaker mechanism [14].

SAN electrical remodelling is a highly coordinated process. Beyond I_f_, the depolarizing drive is further attenuated through a reduction in L- and T-type calcium current densities and Voltage-Gated L-type Calcium Channel Subunit Alpha-1D (Cav1.3) protein expression [9]. A broader dysregulation of sodium and potassium channel gene expression may also contribute to reducing automaticity [8]. A pivotal finding from Girk4-knockout mice (which lack the Acetylcholine-activated Potassium Current (I_KACh_)) revealed that this entire programme of training-induced electrical remodelling—including HCN_4_ and Cav1.3 downregulation—is abolished [9]. This identifies cholinergic signalling, likely from repetitive vagal activation during exercise, as an essential “on-switch” for intrinsic SAN remodelling.

### 4.2. Calcium Clock Remodelling: A Secondary Role

Evidence regarding the remodelling of the “calcium clock” with exercise is less consistent and conclusive than that for the membrane clock. Data across studies are contradictory: while one study in trained rats reported a decrease in Ryanodine Receptor Type 2 (RyR2) mRNA (with only marginally significant protein and functional changes) [8], investigations in mouse models found no significant alterations in the transcripts of key calcium-handling proteins such as RyR2, Sarcoplasmic/Endoplasmic Reticulum Calcium ATPase type 2a (SERCA2a), and phospholamban [8]. These conflicting findings suggest that calcium clock remodelling is not a consistently observed phenomenon, and its role is unclear.

### 4.3. Structural Remodelling and the Pathological Shift

Beyond electrophysiological adaptations, prolonged intense exercise can induce structural alterations that suggest a transition toward pathology, with fibrosis being a key feature in animal models [14,15,18]. In an endurance rat model, significant interstitial collagen deposition and atrial fibrosis were observed, correlating with a markedly increased susceptibility to ventricular tachycardia. This profibrotic state was associated with upregulated Transforming Growth Factor-β1 (TGF-β1) and Matrix Metalloproteinase-2 (MMP-2) at both the transcript and protein levels in the right atrium, alongside increased type I collagen expression [18]. Evidence from a rat swimming model demonstrated SAN ultrastructural injury, including mitochondrial swelling/vacuolization, gap junction loss, and marked Connexin 43 (Cx43) downregulation/lateralization, accompanied by a decrease in intracellular cardiac troponin T(cTnT) that, in contrast to the structural damage, partially reversed after training stopped [15].

The association of right atrial enlargement and ventricular wall thickness [16,19] with sinus node dysfunction in athletes provides a clinical correlate for the structural pathology observed in animal studies, suggesting a common anatomical substrate.

## 5. Moderating Factors in SAN Remodelling

### 5.1. Pronounced Interspecies Disparities

A fundamental divergence exists across species regarding the primary mechanism of training-induced bradycardia. Compelling evidence from humans and certain animal models, such as rabbits and rats, demonstrates substantial intrinsic SAN electrophysiological remodelling, characterized by a lower intrinsic heart rate and the downregulation of key pacemaker ion channels such as HCN_4_ [3,8,10,21,22]. In stark contrast, studies in other models, including mice, horses, and dogs, attribute bradycardia almost entirely to enhanced parasympathetic tone, reporting no consistent intrinsic alterations in SAN function. Some studies in these species even document an increase in HCN_4_ protein levels [4,14].

### 5.2. Training Modality and Cumulative Load

Cohort studies of former professional cyclists and cross-country skiers have revealed a significantly higher incidence of symptomatic bradycardia, sinus arrest, and pacemaker implantation compared to the general population. Notably, former endurance athletes exhibit larger right atrial volumes (29 ± 12 mL vs. 23 ± 8 mL) [12,19]. Among those diagnosed with sinus node dysfunction, the age at diagnosis was significantly higher in strength/mixed-sport athletes (72.4 ± 9.5 years) than in endurance athletes (64.0 ± 7.5 years). Similarly, the age at pacemaker implantation was older in the strength/mixed group (73.0 ± 10.3 years) than in the endurance group (65.0 ± 11.7 years) [13]. Mechanistically, this clinical disparity may arise from differential remodelling pathways. For instance, running-induced resting bradycardia appears to depend primarily on autonomic mechanisms, whereas cycling-induced bradycardia seems to be predominantly mediated by non-autonomic (intrinsic) mechanisms [16]. Variations in training modality (e.g., running vs. swimming) and intensity/duration are likely to influence both the primary cause of exercise-related heart rate reduction and the resulting electrophysiological remodelling phenotype, such as ion channel remodelling (e.g., HCN_4_ downregulation) and calcium clock mechanisms [8,9,10,20].

### 5.3. The Compounding Effect of Age

A critical clinical observation is that athletes with a history of intensive competition require pacemaker implantation for sinus node dysfunction at a significantly earlier age than the general population [11,13], indicating a synergistic interaction between exercise-induced cardiac remodelling and age-related pathological progression.

## 6. Discussion and Perspectives

**Figure 1:**
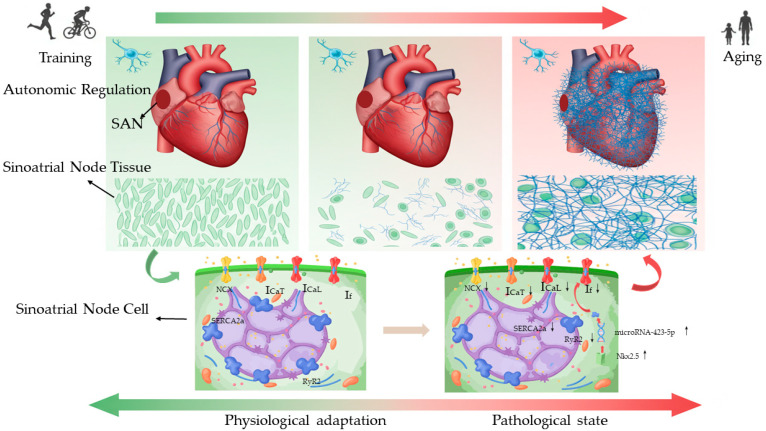
An integrated model of endurance exercise-induced sinoatrial node (SAN) remodelling across the adaptation-pathology continuum.

This systematic review synthesizes evidence from 17 studies on exercise-induced SAN remodelling and its dual nature, encompassing both physiological adaptation and pathological change. Exercise training induces a profound intrinsic electrophysiological reprogramming of the SAN in humans and key animal models that spans a continuum from physiological adaptation to pathology. This sequential trajectory is influenced by species, training patterns, cumulative load, and ageing, ultimately leading to the onset of sinoatrial node disease. Synthesizing the evidence, we present a conceptual framework for exercise-induced SAN remodelling, outlining its progression from an adaptive to a pathological state (Figure 1).

### 6.1. An Ionic Framework: From Adaptation to Pathology

The remodelling of ion channels and the calcium clock, as delineated in this review, provides a mechanistic foundation for both physiological bradycardia and its potential pathological counterparts. Exercise-induced bradycardia is characterized by a coordinated suppression of depolarizing currents: downregulation of I_f_ slows diastolic depolarization [27,28,29,30]; reduced L-type and T-type calcium currents (I_CaL_, I_CaT_) diminish the pacemaker drive [31,32,33]. This may be accompanied by calcium clock dysfunction manifested through impaired sodium–calcium exchanger (NCX), RyR2, and SERCA2a activity, although the evidence here is less consistent [7,33,34]. Further investigation is warranted into the pathways regulated by the ‘membrane clock’ and ‘calcium clock’, such as the Nkx2.5–miR-423-5p regulatory axis and its downregulation of HCN_4_ and I_f_ current [10].

This same framework of ion channel and clock remodelling can be extended to elucidate the pathogenesis of certain forms of sinus tachycardia. In this pathological state, electrical instability may arise from compensatory mechanisms, including the functional upregulation of I_f_, aberrant stimulation of the calcium clock, or a failure of the cholinergic braking system. The pivotal role of the latter is underscored by the Girk4-knockout mouse model, in which the absence of I_KACh_ prevents training-induced bradycardia [9]. Adopting this bidirectional perspective positions the ‘membrane clock’ and ‘calcium clock’ as central, plastic regulators, with clinical SAN dysfunction arising from deviation in either direction from their normal operational range.

### 6.2. Pathological Structural Damage: Structural Remodelling and the Disuse Hypothesis

Beyond intrinsic electrophysiological alterations in sinoatrial node cells, two additional factors may contribute to the development of pathology. First, significant right atrial remodelling—characterized by dilation and fibrosis—has been observed in human athletes and animal models [15,18,19]. Such structural changes may distort SAN architecture and increase impulse propagation resistance [2,35], an effect exacerbated by ageing [36,37,38,39]. These alterations create a substrate conducive to the formation of micro-reentrant circuits, while ectopic escape beats originating within this remodelled tissue can further facilitate the initiation of tachycardia [40]. Secondly, a “disuse” mechanism may be at play. The persistent bradycardic state could lead to long-term suppression of latent, subsidiary pacemaker cells within the SAN that normally possess a higher intrinsic firing rate [41,42]. This suppression, potentially mediated by activity-dependent gene regulation, could gradually reduce their intrinsic automaticity, thereby impairing the functional redundancy and chronotropic reserve of the SAN.

### 6.3. Resolving Disparities and Defining the “Tipping Point”

A critical finding is the profound interspecies disparity in bradycardia mechanisms, which cautions against direct extrapolation from certain large animal models to humans [3,4,10,14,21,22].

Training modality and cumulative load emerge as key moderators, with endurance sports posing the highest risk [13,16]. The finding that I_KACh_ ablation prevents ionic remodelling [9] positions repetitive vagal activation as the “trigger” for long-term molecular reprogramming of the SAN.

To reconcile these findings, we propose the model of “acquired SAN reserve reduction.” We posit that the repeated vagal activation associated with exercise training serves as the primary stimulus that initiates intrinsic electrophysiological remodelling within the SAN. While this remodelling is initially adaptive, producing a beneficial bradycardia, it concurrently reduces the functional reserve of the pacemaker. This process may be accompanied by subtle structural changes. Following detraining, this intrinsic electrophysiological remodelling is reversible, with the accompanying mild structural injury being less completely resolved [15]. In veteran athletes, however, decades of sustained training entrench this acquired state of reduced reserve. When superimposed upon the natural decline in pacemaker function with ageing [43,44,45], the cumulative physiological burden can depress SAN function beyond a critical threshold, thereby precipitating clinical syndromes such as sick sinus syndrome. This model explains the paradoxical observation that highly trained veteran athletes may manifest sinus node dysfunction earlier than their sedentary peers [7,19,46,47].

### 6.4. Clinical Implications and Future Directions

Our analysis leads to two key clinical implications. First, profound bradycardia or sinus pauses in veteran endurance athletes, particularly if symptomatic, require comprehensive evaluation and should not be automatically attributed to a benign athletic adaptation. Second, the interpretation of HRV must be contextualized within the framework of SAN remodelling, recognizing that intrinsic heart rate is a critical determinant of HRV measures [25,26].

Future research should prioritize the following areas: (1) Biomarker Discovery: Identifying circulating or imaging biomarkers that can distinguish adaptive cardiac remodelling from early pathological changes in long-term trained individuals. (2) Longitudinal Athlete Cohorts: Prospectively tracking the remodelling process in veteran athletes using advanced imaging and electro-anatomical mapping to define the natural history of SAN adaptations. (3) Mechanistic Intervention: Exploring mechanism-targeted interventions, such as miR-423-5p inhibition—shown in animal models to reverse training-induced bradycardia [10,48]—as a potential future alternative to pacemaker implantation.

## 7. Limitation

Although a systematic approach was adopted, this review is inherently limited by the constraints of the available published data. First, species differences pose a significant barrier to extrapolating mechanisms across species. Second, the field remains divided on the core mechanisms of bradycardia. This study seeks to reconcile these views but does not fully resolve the underlying disagreement. Furthermore, the evidence for “calcium clock” remodelling is less robust compared to the more established “membrane clock” theory. Finally, due to heterogeneous training practices and a lack of longitudinal evidence, currently no clear dose–response relationship can be defined for the induction of pathological changes.

## 8. Conclusions

In conclusion, current evidence delineates a complex trajectory for the athlete’s sinoatrial node. It is not a question of whether intrinsic electrophysiological remodelling occurs, but under what conditions this physiological adaptation transitions into a pathological state. The path is paved by a coordinated downregulation of the “membrane clock,” a process potentially initiated by repetitive vagal signalling. The progression to clinical disease is likely determined by the superposition of structural alterations within the pacemaker complex and the right atrium, upon which the compounding effects of age and a lifetime of cumulative physiological stress act to exhaust the SAN’s functional reserve. Understanding this multi-step continuum is paramount for identifying at-risk athletes, informing personalized training recommendations, and guiding future therapeutic innovation.

## 9. Methods

A systematic literature search was conducted in accordance with PRISMA guidelines to identify studies on exercise training and sinoatrial node (SAN) electrical remodelling. We searched PubMed, Web of Science Core Collection, and Google Scholar (2000–2025) using a strategy built upon three core domains: exercise intervention, SAN, and electrophysiology/remodelling. Keywords within each domain were combined with “OR”, and the domains were intersected with “AND”. The PubMed search string was: (“exercise training” OR “endurance training” OR “physical conditioning” OR “athletic training”) AND (“sinoatrial node” OR “sinus node” OR “cardiac pacemaker”) AND (“ion channel” OR “electrophysiology” OR “remodelling” OR “bradycardia” OR “arrhythmia”).

Study selection was conducted according to pre-defined eligibility criteria: (1) Population: Human or animal models; (2) Intervention: Structured exercise training; (3) Comparator: Sedentary/untrained controls; (4) Outcomes: Direct measures of SAN ion channel properties or intrinsic electrophysiological function.

**Figure 2:**
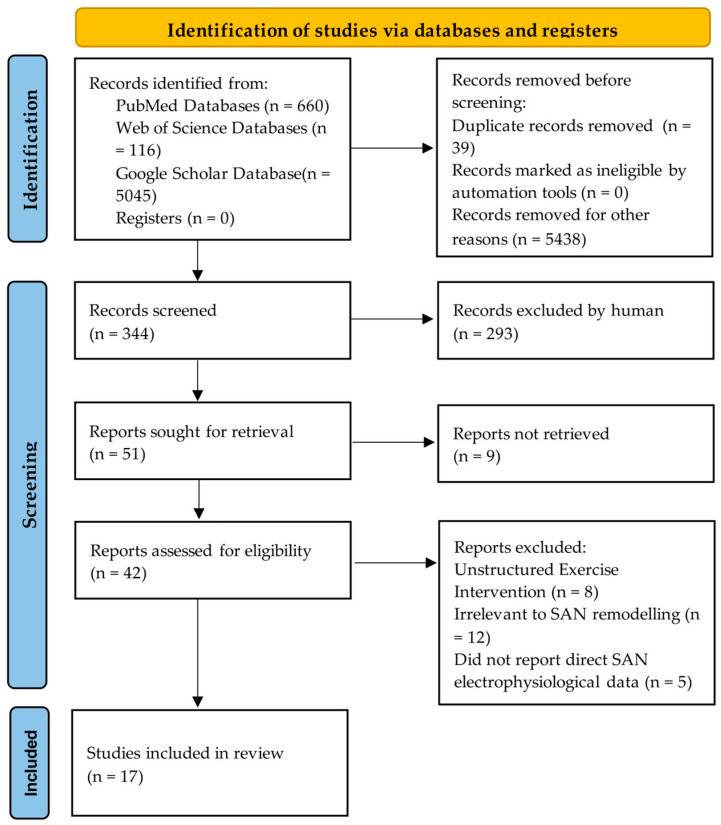
Searches were conducted in PubMed, Web of Science Core Collection, and Google Scholar (2000–2025) following the PRISMA guidelines, resulting in 17 strongly relevant articles.

The selection process followed the PRISMA flow diagram (Figure 2). After duplicate removal, titles/abstracts and full-texts were screened independently by two reviewers. Data from included studies were extracted into a standardized form, with key characteristics and findings synthesized in Table 1.

Critical appraisal of the methodological rigour focused on: (1) experimental design adequacy (e.g., use of control groups); (2) validity of SAN-specific methodologies; and (3) transparency of data and statistical reporting. The implications of these methodological considerations are addressed in the Discussion.

## Figures and Tables

**Table 1:** Intrinsic Remodelling of the Sinus Node with Exercise Training.

Study (Author, Year)	Model (Species)	Exercise Protocol	Major Findings Related to SAN	Electrophysiology Conclusion	Pathological Indicators
Bondarev et al., 2025 [11]	Human	mixed endurance/strength sports	Lifetime endurance sport linked to earlier SAN disease; former athletes required a pacemaker (PM) 5 years earlier (57.9 vs. 64.0 years; *p* = 0.03); borderline inverse correlation between total training load and implantation age (*p* = 0.08); no SAN-size echo differences	Long-term intense sport facilitates earlier manifestation of intrinsic SAN/Atrioventricular Node (AV)-node disease, suggesting cumulative exercise accelerates conduction system ageing rather than a reversible vagal effect	pacemaker implantation
Svedberg et al., 2024 [12]	Human	Long-term endurance training (Skiing)	Male skiers SSS/pacemaker↑ (Heart Rate (HR) 1.19–1.17), younger implant age (63.5 years); no excess in females.	Male endurance athletes SAN dysfunction↑ and pacemaker need; benign post-implant prognosis (sick sinus syndrome is more common)	pacemaker implantation/sick sinus syndrome
Bondarev et al., 2023 [13]	Human	Endurance training, power/mixed training	Endurance athletes received PM ~8 years earlier (65 vs. 73 years, *p* < 0.01); 78% AV-block vs. 44% in power/mixed	Elite endurance training hastens intrinsic SAN/AV-node ageing and early pacemaker requirement	pacemaker implantation
Nissen SD et al., 2022 [4]	Horses	Running	Trained horses had 7% lower resting HR (*p* = 0.001); Sinus Node Recovery Time (SNRT) trended longer at 800 ms Pacing Cycle Length (PCL) (2286 vs. 1927 ms, *p* = 0.09), but no training effect was confirmed	Training lowers resting HR in horses, but SAN adaptation (SNRT prolongation) is modest and non-significant compared with human athletes	Not assessed
Bidaud et al., 2021 [9]	Mouse (WT vs. Girk4^−^/^−^)	Swim training	Training HR↓ 39%, SAN rate 39%; I_f_/I_CaT_/I_CaL_↓, HCN_4_/Cav1.3↓, miR-423-5p↑ (WT only); all blocked in Girk4^−^/^−^	Girk4 deletion blocks miR-HCN_4_/Cav1.3 remodelling, preventing intrinsic bradycardia and athlete’s heart, and offers a therapeutic target for bradycardia	Not assessed
D’Souza et al., 2017 [10]	Human/mice	endurance training/swim training	Athletes: intrinsic HR 11%, ivabradine blunted↓; Mice: miR-423-5p↑8× → HCN4/If↓ (R^2^ = 0.68), SAN rate↓ (R^2^ = 0.46); anti-miR-423 reverses; Nkx2.5↑ drives miR-423-5p transcription → SAN remodelling; upstream master switch	Nkx2.5–miR-423-5p–HCN_4_/I_f_ axis slows SAN rate; anti-miR-423-5p fully rescues	Not assessed
Billman et al., 2015 [14]	Dogs	Running	Resting HR↓ 9%; intrinsic HR/cSNRT unaltered; SAN HCN_4_↑	Bradycardia from ↑parasympathetic; intrinsic SAN unchanged	Cardiac Cavity Fibrosis
Chang et al., 2015 [15]	Rats	Swim training	SAN: collagen, ischemic morphology↑; Cardiac Troponin T (cTnT)/Connexin 43(Cx43)↓; gap-junction loss; swollen mitochondria, endoplasmic reticulum rupture	Repetitive exhaustive exercise → transient SAN ischemia/fibrosis, cTnT/Cx43↓, structural substrate for bradyarrhythmia without permanent failure	SAN collagen deposition, ischemic morphology, Cx43↓, gap junction loss, mitochondrial swelling
D’Souza et al., 2014 [8]	Rats/mice	Running/Swim training	Denervated SAN: cycle↑, I_f_↓ 45%; T-box transcription factor 3 (Tbx3)↓, Neuron-Restrictive Silencer Factor (NRSF)↑, miR-1↑; reversible	Training bradycardia is intrinsic: HCN_4_/I_f_ downregulation via Tbx3↓, NRSF↑, miR-1↑; explains athlete SAN dysfunction	Not assessed
Azevedo et al., 2014 [16]	Human	Running/cycling	Runners vs. cyclists: RHR↓ (45 vs.51 b.min^−1^), vagal↑ (53 vs.41 b.min^−1^), IHR↑ (91 vs.83 b.min^−1^), septal/posterior wall thickness (11 vs. 12 and 11 vs. 12 mm); relationship between IHR and wall thickness r ≈ −0.39.	Sport modality determines bradycardia. Runners: vagal tone; cyclists: vagal tone + SAN remodelling	Not assessed
Molina et al., 2013 [17]	Human	Bike training	Athletes: resting HR 50 vs. 63 bpm (*p* = 0.0004); no HRV index differed (*p* = 0.17–0.97) except trend to lower LF-power	Bradycardia in cyclists is independent of altered autonomic modulation; it likely reflects intrinsic SAN adaptation rather than vagal dominance	Not assessed
Benito et al., 2011 [18]	Rats	Running	QRS duration↑ (ventricular conduction delay), atrial fibrosis↑, Transforming Growth Factor-beta 1 (TGF-β1)↑, Matrix Metalloproteinase-2 (MMP-2)↑, collagen-I↑ in Right Atrium/Left Atrium/Right Ventricle, inducible Ventricular Tachycardia in 42% of trained rats vs. 6% in sedentary	Long-term intensive exercise induces atrial and Right ventricular fibrosis, alters conduction, and increases arrhythmia susceptibility; changes are reversible after detraining	Right atrial fibrosis,↑ TGF-β1↑, MMP-2↑, collagen-I↑
Baldesberger et al., 2008 [19]	Human	cycling	Athletes: ventricular tachycardias↑ (15 vs. 3%), HR↓ (66 vs. 70 bpm), Sinus Node Dysfunction (SND)↑ (10 vs. 2%), pacemaker for bradyarrhythmias (3 vs. 0%), maxRR↑ (6 vs. 0%)	Extreme endurance → lifelong SND/brady risk↑; irreversible SAN remodelling	pacemaker implantation/larger right atrial volume (29 ± 12 mL vs. 23 ± 8 mL)
De Angelis et al., 2004 [20]	Mouse	Running	Trained vs. sedentary: HR↓ (485 vs. 612 bpm); vagal effect (methylatropine) effect↑ (139 vs. 40 bpm), sympathetic effect (propranolol) effect (49 vs. 97 bpm); not intrinsic HR change	Training-induced mouse bradycardia is mediated solely by enhanced cardiac vagal tone and reduced sympathetic drive, without intrinsic SAN remodelling	Not assessed
Stein et al., 2002 [21]	Human	Running	Athletes vs. nonathletes: Sinus Cycle Length (SCL) (before/after block)↑ (1030 vs. 913 ms/737 vs. 653 43 ms), SNRT/SCL (before/after parasympathetic blockade, after double-autonomic blockade)↑ (1.36 vs. 1.26/0.06 vs. 1.45/0.09 vs. 1.31); indicating intrinsic SAN adaptation.	Athletes: SAN remodelling and AV node conduction changes	Not assessed
Such et al., 2002 [22]	Rabbits	Running	Trained vs. untrained: R-R↑28% (365 vs. 286 ms), SNRT↑28% (554 vs. 460 ms); cSNRT/SACT unchanged	Training intrinsically slows SAN rate and recovery, independent of autonomic, structural, or vascular factors—direct electrophysiological remodelling	Not assessed
Stein et al., 2000 [23]	Human	Running	Athletes vs. untrained: cSNRT↑ (369 vs. 279 ms, *p* = 0.09); Wenckebach point↓ (*p* = 0.01); vagal tone correlates with AV delay (r = 0.48)	Vagal tone/intrinsic adaptations of the conduction system: SAN automaticity slightly reduced; AV delay more marked	Not assessed

↑: increased; ↓: decreased.

## Data Availability

No new data were created or analyzed in this study. Data sharing is not applicable to this article.

## Abbreviations

The following abbreviations are used in this manuscript:

AV	Atrioventricular Node
Cav1.3	Voltage-Gated L-type Calcium Channel Subunit Alpha-1D
Cx43	Connexin 43
cTnT	Cardiac Troponin T
HCN	Hyperpolarization-activated Cyclic Nucleotide-gated Channel
HCN_4_	Hyperpolarization-activated Cyclic Nucleotide-gated Channel 4
HR	Heart Rate
HRV	Heart Rate Variability
I_CaL_	L-type Calcium Current
I_CaT_	T-type Calcium Current
I_f_	Hyperpolarization-activated Cyclic Nucleotide-Gated Cation Channel
I_KACh_	Acetylcholine-activated Potassium Current
MMP-2	Matrix Metalloproteinase-2
NRSF	Neuron-Restrictive Silencer Factor
NCX	Sodium-Calcium Exchanger
PCL	Pacing Cycle Length
PM	PaceMaker
RyR2	Ryanodine Receptor Type 2
SAN	Sinoatrial Node
SERCA2a	Sarcoplasmic/Endoplasmic Reticulum Calcium ATPase type 2a
SCL	Sinus Cycle Length
SNRT	Sinus Node Recovery Time
Tbx3	T-box Transcription Factor 3
TGF-β1	Transforming Growth Factor-β1

## References

[1] Fagard R. (2003). Athlete’s heart. Heart (Br. Card. Soc.).

[2] O’Keefe J.H., Patil H.R., Lavie C.J., Magalski A., Vogel R.A., McCullough P.A. (2012). Potential adverse cardiovascular effects from excessive endurance exercise. Mayo Clin. Proc..

[3] Boyett M.R., D’Souza A., Zhang H., Morris G.M., Dobrzynski H., Monfredi O. (2013). Viewpoint: Is the resting bradycardia in athletes the result of remodeling of the sinoatrial node rather than high vagal tone?. J. Appl. Physiol..

[4] Nissen S.D., Weis R., Krag-Andersen E.K., Hesselkilde E.M., Isaksen J.L., Carstensen H., Kanters J.K., Linz D., Sanders P., Hopster-Iversen C. (2022). Electrocardiographic characteristics of trained and untrained standardbred racehorses. J. Vet. Intern. Med..

[5] Hawks M.K., Paul M.L.B., Malu O.O. (2021). Sinus Node Dysfunction. Am. Fam. Physician.

[6] Bashour T.T. (1985). Classification of sinus node dysfunction. Am. Heart J..

[7] Choudhury M., Boyett M.R., Morris G.M. (2015). Biology of the Sinus Node and its Disease. Arrhythmia Electrophysiol. Rev..

[8] D’Souza A., Bucchi A., Johnsen A.B., Logantha S.J., Monfredi O., Yanni J., Prehar S., Hart G., Cartwright E., Wisloff U. (2014). Exercise training reduces resting heart rate via downregulation of the funny channel HCN4. Nat. Commun..

[9] Bidaud I., D’Souza A., Forte G., Torre E., Greuet D., Thirard S., Anderson C., Chung You Chong A., Torrente A.G., Roussel J. (2020). Genetic Ablation of G Protein-Gated Inwardly Rectifying K_+_ Channels Prevents Training-Induced Sinus Bradycardia. Front. Physiol..

[10] D’Souza A., Pearman C.M., Wang Y., Nakao S., Logantha S., Cox C., Bennett H., Zhang Y., Johnsen A.B., Linscheid N. (2017). Targeting miR-423-5p Reverses Exercise Training-Induced HCN4 Channel Remodeling and Sinus Bradycardia. Circ. Res..

[11] Bondarev S., Brotto L., Graziano F., Cipriani A., Corrado D., Zorzi A. (2025). Does Long-Term Sport Practice Facilitate the Development of Idiopathic Bradycardia Requiring Early Pacemaker Implantation During the Course of Life?. J. Cardiovasc. Dev. Dis..

[12] Svedberg N., Sundström J., James S., Hållmarker U., Hambraeus K., Andersen K. (2024). Long-Term Incidence of Bradycardia and Pacemaker Implantations Among Cross-Country Skiers: A Cohort Study. Circulation.

[13] Bondarev S., Achkasov E., Zorzi A., Safaryan A., Graziano F., Sizov A. (2023). Intrinsic Sinus Node/Atrioventricular Node Dysfunction Requiring Pacemaker Implantation: Role of Former Professional Sport Activity. J. Clin. Med..

[14] Billman G.E., Cagnoli K.L., Csepe T., Li N., Wright P., Mohler P.J., Fedorov V.V. (2015). Exercise training-induced bradycardia: Evidence for enhanced parasympathetic regulation without changes in intrinsic sinoatrial node function. J. Appl. Physiol..

[15] Chang Y., Yu T., Yang H., Peng Z. (2015). Exhaustive exercise-induced cardiac conduction system injury and changes of cTnT and Cx43. Int. J. Sports Med..

[16] Azevedo L.F., Perlingeiro P.S., Hachul D.T., Gomes-Santos I.L., Brum P.C., Allison T.G., Negrão C.E., De Matos L.D. (2014). Sport modality affects bradycardia level and its mechanisms of control in professional athletes. Int. J. Sports Med..

[17] Molina G.E., Porto L.G., Fontana K.E., Junqueira L.F. (2013). Unaltered R-R interval variability and bradycardia in cyclists as compared with non-athletes. Clin. Auton. Res. Off. J. Clin. Auton. Res. Soc..

[18] Benito B., Gay-Jordi G., Serrano-Mollar A., Guasch E., Shi Y., Tardif J.C., Brugada J., Nattel S., Mont L. (2011). Cardiac arrhythmogenic remodeling in a rat model of long-term intensive exercise training. Circulation.

[19] Baldesberger S., Bauersfeld U., Candinas R., Seifert B., Zuber M., Ritter M., Jenni R., Oechslin E., Luthi P., Scharf C. (2008). Sinus node disease and arrhythmias in the long-term follow-up of former professional cyclists. Eur. Heart J..

[20] De Angelis K., Wichi R.B., Jesus W.R., Moreira E.D., Morris M., Krieger E.M., Irigoyen M.C. (2004). Exercise training changes autonomic cardiovascular balance in mice. J. Appl. Physiol..

[21] Stein R., Medeiros C.M., Rosito G.A., Zimerman L.I., Ribeiro J.P. (2002). Intrinsic sinus and atrioventricular node electrophysiologic adaptations in endurance athletes. J. Am. Coll. Cardiol..

[22] Such L., Rodriguez A., Alberola A., Lopez L., Ruiz R., Artal L., Pons I., Pons M.L., García C., Chorro F.J. (2002). Intrinsic changes on automatism, conduction, and refractoriness by exercise in isolated rabbit heart. J. Appl. Physiol..

[23] Stein R., Moraes R.S., Cavalcanti A.V., Ferlin E.L., Zimerman L.I., Ribeiro J.P. (2000). Atrial automaticity and atrioventricular conduction in athletes: Contribution of autonomic regulation. Eur. J. Appl. Physiol..

[24] al-Ani M., Munir S.M., White M., Townend J., Coote J.H. (1996). Changes in R-R variability before and after endurance training measured by power spectral analysis and by the effect of isometric muscle contraction. Eur. J. Appl. Physiol. Occup. Physiol..

[25] Monfredi O., Lyashkov A.E., Johnsen A.B., Inada S., Schneider H., Wang R., Nirmalan M., Wisloff U., Maltsev V.A., Lakatta E.G. (2014). Biophysical characterization of the underappreciated and important relationship between heart rate variability and heart rate. Hypertension.

[26] Bai X., Wang K., Yuan Y., Li Q., Dobrzynski H., Boyett M.R., Hancox J.C., Zhang H. (2017). Mechanism underlying impaired cardiac pacemaking rhythm during ischemia: A simulation study. Chaos.

[27] Ludwig A., Budde T., Stieber J., Moosmang S., Wahl C., Holthoff K., Langebartels A., Wotjak C., Munsch T., Zong X. (2003). Absence epilepsy and sinus dysrhythmia in mice lacking the pacemaker channel HCN2. EMBO J..

[28] Yamamoto M., Dobrzynski H., Tellez J., Niwa R., Billeter R., Honjo H., Kodama I., Boyett M.R. (2006). Extended atrial conduction system characterised by the expression of the HCN4 channel and connexin45. Cardiovasc. Res..

[29] Shi W., Wymore R., Yu H., Wu J., Wymore R.T., Pan Z., Robinson R.B., Dixon J.E., McKinnon D., Cohen I.S. (1999). Distribution and prevalence of hyperpolarization-activated cation channel (HCN) mRNA expression in cardiac tissues. Circ. Res..

[30] Nikmaram M.R., Boyett M.R., Kodama I., Suzuki R., Honjo H. (1997). Variation in effects of Cs+, UL-FS-49, and ZD-7288 within sinoatrial node. Am. J. Physiol..

[31] Kodama I., Nikmaram M.R., Boyett M.R., Suzuki R., Honjo H., Owen J.M. (1997). Regional differences in the role of the Ca^2+^ and Na^+^ currents in pacemaker activity in the sinoatrial node. Am. J. Physiol..

[32] Hagiwara N., Irisawa H., Kameyama M. (1988). Contribution of two types of calcium currents to the pacemaker potentials of rabbit sino-atrial node cells. J. Physiol..

[33] Tellez J.O., Dobrzynski H., Greener I.D., Graham G.M., Laing E., Honjo H., Hubbard S.J., Boyett M.R., Billeter R. (2006). Differential expression of ion channel transcripts in atrial muscle and sinoatrial node in rabbit. Circ. Res..

[34] Bogdanov K.Y., Vinogradova T.M., Lakatta E.G. (2001). Sinoatrial nodal cell ryanodine receptor and Na^+^-Ca^2+^ exchanger: Molecular partners in pacemaker regulation. Circ. Res..

[35] Kohl P., Hunter P., Noble D. (1999). Stretch-induced changes in heart rate and rhythm: Clinical observations, experiments and mathematical models. Prog. Biophys. Mol. Biol..

[36] Easterling M., Rossi S., Mazzella A.J., Bressan M. (2021). Assembly of the Cardiac Pacemaking Complex: Electrogenic Principles of Sinoatrial Node Morphogenesis. J. Cardiovasc. Dev. Dis..

[37] Lev M. (1954). Aging changes in the human sinoatrial node. J. Gerontol..

[38] James T.N. (1961). Anatomy of the human sinus node. Anat. Rec..

[39] Glukhov A.V., Kalyanasundaram A., Lou Q., Hage L.T., Hansen B.J., Belevych A.E., Mohler P.J., Knollmann B.C., Periasamy M., Györke S. (2015). Calsequestrin 2 deletion causes sinoatrial node dysfunction and atrial arrhythmias associated with altered sarcoplasmic reticulum calcium cycling and degenerative fibrosis within the mouse atrial pacemaker complex1. Eur. Heart J..

[40] Allessie M., Ausma J., Schotten U. (2002). Electrical, contractile and structural remodeling during atrial fibrillation. Cardiovasc. Res..

[41] Brorson L., Conradson T.B., Olsson B., Varnauskas E. (1976). Right atrial monophasic action potential and effective refractory periods in relation to physical training and maximal heart rate. Cardiovasc. Res..

[42] Kodama I., Boyett M.R. (1985). Regional differences in the electrical activity of the rabbit sinus node. Pflug. Arch. Eur. J. Physiol..

[43] Jones S.A., Boyett M.R., Lancaster M.K. (2007). Declining into failure: The age-dependent loss of the L-type calcium channel within the sinoatrial node. Circulation.

[44] Larson E.D., St Clair J.R., Sumner W.A., Bannister R.A., Proenza C. (2013). Depressed pacemaker activity of sinoatrial node myocytes contributes to the age-dependent decline in maximum heart rate. Proc. Natl. Acad. Sci. USA.

[45] Boyett M.R., Yanni J., Tellez J., Bucchi A., Mesirca P., Cai X., Logantha S., Wilson C., Anderson C., Ariyaratnam J. (2021). Regulation of sinus node pacemaking and atrioventricular node conduction by HCN channels in health and disease. Prog. Biophys. Mol. Biol..

[46] Northcote R.J., Canning G.P., Ballantyne D. (1989). Electrocardiographic findings in male veteran endurance athletes. Br. Heart J..

[47] Dobrzynski H., Boyett M.R., Anderson R.H. (2007). New insights into pacemaker activity: Promoting understanding of sick sinus syndrome. Circulation.

[48] Petkova M., Atkinson A.J., Yanni J., Stuart L., Aminu A.J., Ivanova A.D., Pustovit K.B., Geragthy C., Feather A., Li N. (2020). Identification of Key Small Non-Coding MicroRNAs Controlling Pacemaker Mechanisms in the Human Sinus Node. J. Am. Heart Assoc..

